# Commentary: Deficient inhibition in alcohol-dependence: let's consider the role of the motor system!

**DOI:** 10.3389/fnins.2019.01237

**Published:** 2019-11-15

**Authors:** Raffaele Nardone, Eugen Trinka, Luca Sebastianelli, Viviana Versace, Leopold Saltuari

**Affiliations:** ^1^Department of Neurology, Franz Tappeiner Hospital, Merano, Italy; ^2^Department of Neurology, Christian Doppler Klinik, Paracelsus Medical University, Salzburg, Austria; ^3^Karl Landsteiner Institut für Neurorehabilitation und Raumfahrtneurologie, Salzburg, Austria; ^4^Centre for Cognitive Neurosciences Salzburg, Salzburg, Austria; ^5^University for Medical Informatics and Health Technology, UMIT, Hall in Tirol, Austria; ^6^Department of Neurorehabilitation, Hospital of Vipiteno, Vipiteno, Italy; ^7^Research Unit for Neurorehabilitation South Tyrol, Bolzano, Italy; ^8^Department of Neurology, Hochzirl Hospital, Zirl, Austria

**Keywords:** alcohol-dependence, inhibition, cortical excitability, gabaergic, glutamatergic, transcranial magnetic stimulation

We have read with great interest the commentary “Deficient inhibition in alcohol-dependence: let's consider the role of the motor system!” (Zhou et al., 2019), recently published in this Journal, on the manuscript “Deficient inhibition in alcohol-dependence: let's consider the role of the motor system!” published in 2018 in “Neuropsychopharmacology” (Quoilin et al., 2018).

In their interesting study, Quoilin et al. tested the hypothesis that appropriate neural inhibition of the motor output pathways is altered in alcohol-dependence (AD). During an instructed-delay choice reaction time task, suppression of the motor evoked potentials (MEP) elicited by transcranial magnetic stimulation (TMS) in the delay period relative to baseline for the forthcoming movement was significantly weaker in subjects with AD than in healthy control subjects, thus suggesting a storage of neural inhibition in AD patients. In their commentary, Zhou and colleagues highlighted the role of the motor system in the deficient inhibitory control.

The importance of neural inhibitory mechanisms at motor cortical level in subjects with AD clearly emerges from both articles. In fact inhibitory, mainly GABAergic, transmission plays a key role in the neurochemical mechanisms on the basis of intoxication, tolerance and withdrawal (Koob, 2004).

However, altered motor cortical excitability may be caused by a dysfunction in the neural inhibitory circuits, but also by an impairment of the intracortical excitatory circuits.

Quite surprisingly, in both papers the role of excitatory, mainly glutamatergic, neurotransmission in AD has not been specifically considered.

Indeed, ethanol abuse also affects the central nervous system by altering the function also of excitatory transmission (Rudolph et al., 1997; Harris et al., 2003), resulting in reduced overall brain excitability. Acute ethanol intake enhances the effects of GABA on GABA_A_ receptors and inhibits glutamatergic function by decreasing cationic conductance through the ionotropic type of glutamate receptors. Chronic alcohol exposure appears to create inverse changes in the functions of these systems leading to decreased GABAergic and increased glutamatergic functions bringing about the development of tolerance and/or physical dependence on alcohol (Littleton, 2001).

Several cell and animal studies (Di Chiara et al., 1998; Nagy et al., 2005) suggest that the glutamatergic system is an especially important factor in the mediation of the addictive effect of alcohol. In particular, the N-methyl-d-aspartate (NMDA) receptors exhibit the highest affinity targets for ethanol in the CNS (Lovinger et al., 1990; Grant and Lovinger, 1995; Hoffman and Tabakoff, 1996).

Transcranial magnetic stimulation (TMS) can be applied in different paradigms to obtain a measure of various aspects of cortical excitability. The different TMS paradigms provide information about different neurotransmitter systems and neurochemical pathways (Hallett, 2000; Rossini et al., 2015). In particular, TMS given in a paired-pulse paradigm allows the assessment of the intracortical facilitatory and inhibitory circuits that influence the cortical motor output (Paulus et al., 2008; Groppa et al., 2012).

A TMS study demonstrated that chronic ethanol abuse alters glutamate-dependent mechanisms of short-term cortical plasticity (Conte et al., 2008). Interestingly, another TMS study showed a selective increase in intracortical facilitation to paired TMS (Nardone et al., 2010) in AD and alcohol withdrawal. This parameter is thought to depend upon the activity of intracortical glutamatergic circuits (Tokimura et al., 1996; Liepert et al., 1997; Ziemann et al., 1998; Di Lazzaro et al., 1999; Reis et al., 2006).

Prolonged ethanol exposition leads to a compensatory “upregulation” of NMDA receptors mediated functions, which is thought to play a crucial role in the occurrence of ethanol tolerance and dependence.

Therefore, AD is characterized by a motor cortical hyperexcitability, which can be secondary to an increased glutamatergic action rather than to a reduced GABAergic activity. Anti-glutamatergic approaches could thus represent an efficacious and preferable alternative for treating AD and alcohol withdrawal symptoms.

On the other hand, not only GABA and glutamate, but many other neurotransmitters and neuromodulators (De Witte et al., 2003) can be involved in the complex system of AD neurobiology.

As pointed out by Zhou in the above-mentioned commentary, deficient neural motor inhibition can serve as objective TMS-based biomarker, which help to detect people at high-risk of alcohol relapse, and also represent a promising target for pharmacological and training interventions. Therefore, it is of crucial importance to correctly identify and define the mechanisms of the impaired inhibition in AD, and the role of the concomitant enhanced glutamatergic transmission cannot be overlooked.

## Author Contributions

RN conceived the idea and wrote the manuscript. ET read and revised the manuscript. LSe, VV, and LSa revised the literature and wrote the manuscript.

### Conflict of Interest

The authors declare that the research was conducted in the absence of any commercial or financial relationships that could be construed as a potential conflict of interest.
